# Hypothyroidism and Adverse Endpoints in Diabetic Patients: A Systematic Review and Meta-Analysis

**DOI:** 10.3389/fendo.2019.00889

**Published:** 2020-01-10

**Authors:** Shaojun Zhang, Guilong Feng, Fangfang Kang, Yali Guo, Hongyan Ti, Lufang Hao, Peng Gao, Jiangqin Gao

**Affiliations:** ^1^Department of Endocrinology, Shanxi Province People's Hospital, Taiyuan, China; ^2^Department of Endocrinology, The Sixth Division Hospital of Xinjiang Production and Construction Corps, Wujiaqu, China; ^3^Department of Emergency, The First Hospital of Shanxi Medical University, Taiyuan, China; ^4^The Department of Endocrinology, The Central Hospital of Linfen City, Linfen, China; ^5^The Department of Digestive, Linfen People's Hospital, Linfen, China

**Keywords:** thyroid function, adverse endpoints, diabetes, meta-analysis, cardiovascular event

## Abstract

**Background:** This study investigated the relationship strength between hypothyroidism and cardiovascular and renal outcomes in diabetic patients.

**Methods:** The electronic databases PubMed, EmBase, and Cochrane library were screened for relevant studies published before November 2018. The outcomes included major cardiovascular events (MACEs), all-cause mortality, cardiac death, stroke, diabetic nephropathy (DN), diabetic retinopathy (DR), and chronic kidney disease (CKD). The pooled results for all outcomes were calculated using random-effects models.

**Results:** A total of eight studies met the inclusion criteria. The summary results indicated that hypothyroidism was not associated with the risk of MACEs (OR:1.21; 95%CI:0.68–2.16; *P* = 0.514), all-cause mortality (OR:1.27; 95%CI:0.93–1.74; *P* = 0.136), cardiac death (OR:1.16; 95%CI:0.89–1.52; *P* = 0.271), stroke (OR:0.96; 95%CI: 0.49–1.88; *P* = 0.915), and DN (OR:1.71; 95%CI:0.37–7.90; *P* = 0.490). There was a significant association between hypothyroidism and the risk of DR (OR:1.73; 95%CI:1.08–2.77; *P* = 0.023) and CKD (OR:1.22; 95%CI:1.10–1.36; *P* < 0.001).

**Conclusions:** These findings indicate that diabetic patients with hypothyroidism have an increased risk of DR and CKD. Additional large-scale prospective studies should be carried out to verify the prognosis of patients with diabetes and hypothyroidism.

## Introduction

Diabetes mellitus (DM) is a metabolic disorder with a gradually increasing prevalence, accounting for 451 million people worldwide in 2017, and this prevalence is expected to increase to 693 million by 2045. Nearly half of the individuals (49.7%) with diabetes might be undiagnosed (1). Diabetic patients have high blood glucose levels and are at increased risk of several serious life-threatening health conditions, leading to high medical care costs, poor quality of life, and high mortality risk (2). Elevated blood glucose could induce vascular damage leading to injuries to the heart, eyes, kidneys, and nerves (3). Therefore, improving the complication status of diabetic patients has become imperative, and a healthy lifestyle is widely used to reach this goal.

Patients with hypothyroidism have high levels of thyroid-stimulating hormone (TSH) and normal free T4 levels, accounting for nearly 9% of the adults and 17% of the diabetic patients (4, 5). The differences in the prevalence between diabetic patients and the general population could be due to race, age, sex, body mass index (BMI), dietary iodine intake, and serum TSH diagnostic cutoff values (6). Although there might be few signs and symptoms of thyroid dysfunction in patients with hypothyroidism, the clinical, endocrine, and metabolic changes could affect the prognosis of the patients (7). Moreover, hypothyroidism is associated with impaired cardiac function and the progression of atherosclerosis and myocardial dysfunction (8, 9). Nevertheless, whether the treatment of hypothyroidism in diabetic patients is warranted still remains an important debate.

Given the high prevalence of hypothyroidism among diabetic patients, the management of hypothyroidism is important to improve the prognosis of diabetes. No systematic reviews and meta-analyses were conducted to date regarding the correlation between hypothyroidism and diabetic complications. Therefore, the present study was conducted to provide a quantitative pooled correlation between hypothyroidism and diabetic complications.

## Methods

### Data Sources, Search Strategy, and Selection Criteria

This systematic review and meta-analysis was conducted and reported according to the Preferred Reporting Items for Systematic Reviews and Meta-Analysis (PRISMA) Statement issued in 2009 (Checklist S1) (10). We systematically searched the electronic databases PubMed, EmBase, and Cochrane library for the studies that were published by November 2018 regarding the association of hypothyroidism with diabetic complications. The keywords were used in combination with MeSH and text words: (“diabetes mellitus” OR diabetes OR diabetic) AND (“hypothyroidism” OR hypothyroidism) AND (“coronary heart diseases” OR “ischemic heart diseases” OR “myocardial infarction” OR “coronary atherosclerosis” OR “cardiovascular diseases” OR “death” OR “mortality” OR “heart failure” OR “stroke” OR “atrial fibrillation” OR “arrhythmia” OR “peripheral artery diseases”). The reference lists from the relevant original studies and reviews were manually searched to identify any additional eligible study. Ethical approval and informed consent are not applicable to meta-analyses.

The study selection process was conducted by two authors, and any disagreement was resolved by group discussion until a consensus was reached. A study was included if it met the following inclusion criteria: (1) Study design: observational study, irrespective of cross-sectional, retrospective, or prospective design. (2) Patients: all patients diagnosed with diabetes. (3) Exposure: patients with hypothyroidism. (4) Control: patients with euthyroidism. (5) Outcomes: the study had to report at least one of the following outcomes: major cardiovascular events (MACEs), all-cause mortality, cardiac death, stroke, diabetic nephropathy (DN), diabetic retinopathy (DR), and chronic kidney disease (CKD). Case reports, case series, reviews, *in vitro* studies, or *in vivo* experiments were excluded de facto.

### Data Collection and Quality Assessment

Two authors independently reviewed the full text of the studies for rigorous data collection, using a standard data extraction form. Any disagreement was resolved by discussion, or further evaluation was conducted by the corresponding author. The data collected included first author's name, publication year, study design, sample size, number of hypothyroidism patients, the TSH cutoff values that defined hypothyroidism, mean age, percentage of male sex, current smoker, BMI, disease status, DM duration, reported outcomes, and adjusted factors. The study quality was evaluated using the Newcastle-Ottawa Scale (NOS), which is based on selection (four items), comparability (one item), and outcome (three items) (11). The quality assessment was carried out by two authors. Any inconsistencies were settled by group discussion by referring to the original study until a level of 95% agreement was reached.

### Statistical Analysis

All analyses were carried out using the STATA software (version 10.0; Stata Corporation, College Station, TX, USA). The prognostic role of hypothyroidism in diabetic patients was calculated based on the effect estimates and 95% confidence intervals (CIs) in individual studies. The summary of the odds ratios (ORs) and 95%CIs were calculated using random-effects models for hypothyroidism vs. euthyroidism (12, 13). The I^2^-and *P*-values for Q statistics were used to calculate the heterogeneity among the included studies (14, 15). Sensitivity analyses were performed for MACEs and all-cause mortality to evaluate the influence of each study in the meta-analysis (16). Subgroup analyses for MACEs and all-cause mortality were conducted based on study design, country, percentage of male sex, DM type, and study quality. *P*-values between subgroups were also calculated to evaluate the differences among the subgroups (17). Publication biases for MACEs and all-cause mortality were calculated using funnel plots, Egger tests (18), and Begg tests (19). All other reported *P*-values were two-sided, and *P* < 0.05 were considered to be statistically significant for all included studies.

## Results

### Literature Search

**Figure 1:**
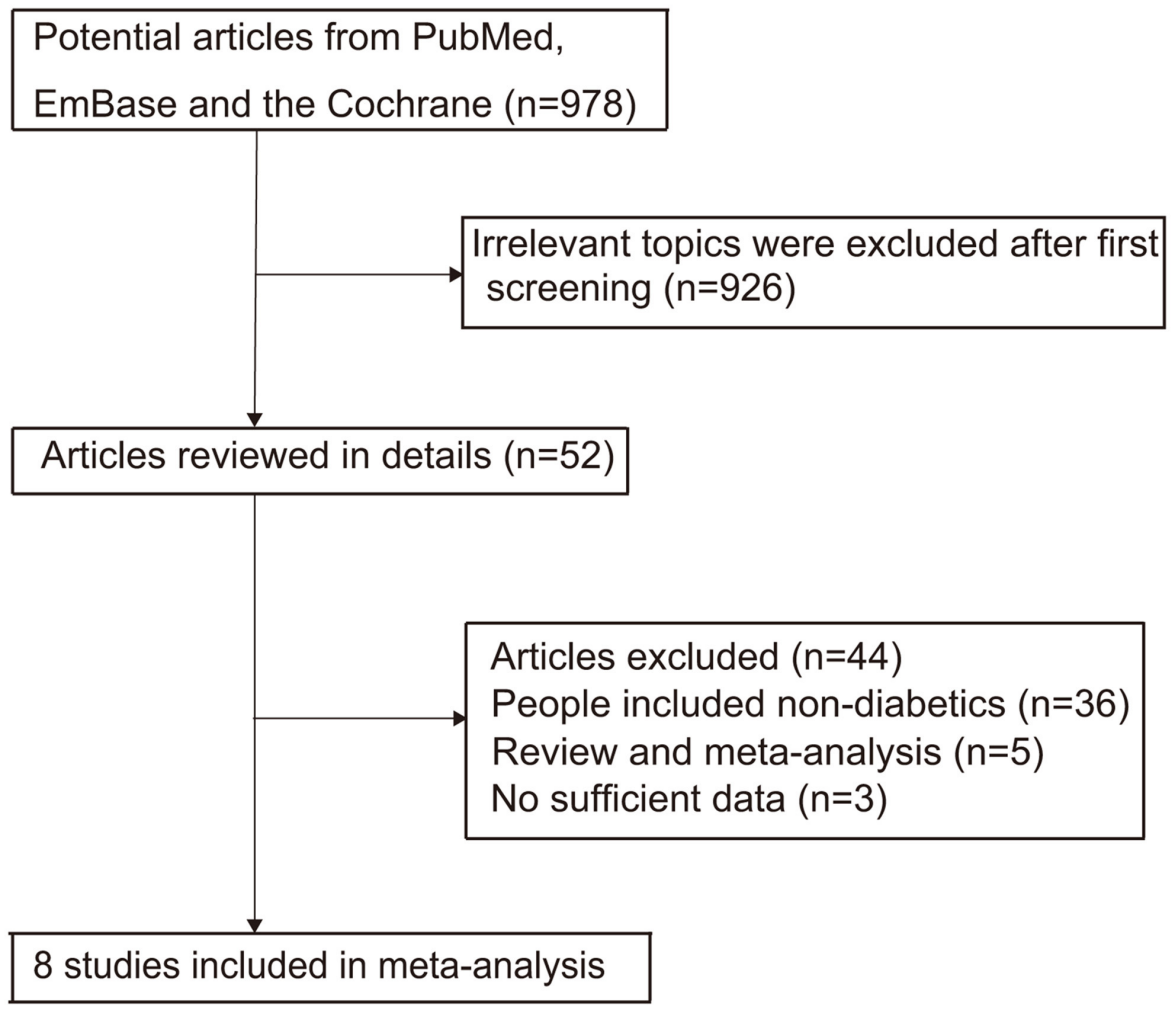
The flowchart of the study selection process.

The literature screening process is summarized in Figure 1. The initial search in PubMed, EmBase, and Cochrane library retrieved 978 studies that were potentially relevant. Then, 926 studies were excluded due to an irrelevant topic or duplications after reviewing the titles and abstracts. Subsequently, 52 potentially eligible studies were evaluated, and 44 studies were excluded due to the following reasons: patients were not diagnosed with diabetes (*n* = 36), review or meta-analysis (*n* = 5), and not the desired outcomes (*n* = 3). Manual search of the reference lists of these relevant studies did not yield any new eligible studies. Finally, eight studies were selected for the meta-analysis (20–27). The baseline characteristics of the included studies are summarized in Table 1.

**Table 1:** Baseline characteristic of studies included in the systematic review and meta-analysis.

**Study**	**Publication year**	**Study design**	**Country**	**Sample size**	**Number of patients with hypothyroidism**	**The TSH cutoff values that defined hypothyroidism**	**Mean age (years)**	**Percentage male (%)**	**Current smoker (%)**	**BMI (Kg/m^**2**^)**	**Disease status**	**DM duration (years)**	**Reported outcomes**	**Adjusted factors**	**NOS score**
Chen et al. (20)	2007	Cross- sectional	China	556	41	4.0–20.0 mU/l	66.4	64.9	20.8	26.1	Type 2 DM	9.6	DR, DN, MACE, cardiac death, All-cause mortality	Age, sex, HbA 1c, total cholesterol, HDLcholesterol, BP, BMI, smoking status, medication andurinary albumin, creatinine excretion	7
Drechsler et al. (23)	2014	Prospective	Germany	988	16	4.1–15.0 mU/l	65.6	53.7	40.4	27.6	Type 2 DM	18.3	MACE, stroke, cardiac death, All-cause mortality	Age, sex, atorvastatin, systolic BP, BMI, left ventricular hypertrophy, albuminlevel, creatinine level, N-terminal pro2-B-type natriuretic peptide level, and ultrafiltration volume	8
Sathyapalan et al. (21)	2010	Retrospective	UK	944	472	NS	72.1	50.9	NS	NS	Type 2 DM	NS	MACE, cardiac death, All-cause mortality	Age, sex, or the other covariates	7
Jia et al. (24)	2015	Cross-sectional	China	933	126	>5.0 mU/l	61.8	46.8	29.1	24.8	Type 2 DM	7.3	MACE, stroke, and CKD	Age, gender, diabetes duration, hypertension, smoking and drinking status, BMI and HbA 1c	8
Lin et al. (27)	2017	Retrospective	China	2332	478	NS	65.8	29.3	NS	NS	NS	NS	All-cause mortality	Sex, age, and comorbidities	6
Journy et al. (25)	2017	Prospective	US	NS	NS	NS	NS	0.0	NS	NS	NS	NS	All-cause mortality	Baseline year and age, race/ethnicity, BMI, family history of breast cancer, and life-style and reproductive factors	6
Kim et al. (22)	2011	Retrospective	Korea	489	61	>4.0 mU/l	58.3	45.6	NS	24.7	Type 2 DM	7.1	DN, DR	Age, gender, BMI, HbA1c, duration of DM, hypertension	7
Zhou et al. (26)	2017	Retrospective	China	3815	545	4.78–19.9 mU/l	56.4	42.4	NS	25.2	Type 2 DM	7.7	CKD	Age, duration of DM, HbA1C, BMI, BP, and LDL cholesterol	7

*BMI, body mass index; BP, blood pressure; CKD, chronic kidney disease; DM, diabetes mellitus; DN, diabetic nephropathy; DR, diabetic retinopathy; MACE, major cardiovascular events; MI, myocardial infarction; NS, not specified; TSH, thyroid-stimulating hormone.

### Study Characteristics

Of the eight included studies, two had a cross-sectional design, two had a prospective cohort design, and the remaining four studies had a retrospective cohort design. Five studies were conducted in Eastern countries, and the remaining three studies were conducted in Western countries. The mean age of the included patients ranged from 56.4 to 72.1 years, and the percentage of male sex ranged from 0.0 to 64.9%. Six studies included patients with type 2 DM, and the remaining two studies did not specify the type of DM. Study quality was assessed using NOS, and two studies obtained 8 stars, four obtained 7 stars, and the remaining two obtained 6 stars.

### Major Cardiovascular Events

**Figure 2:**
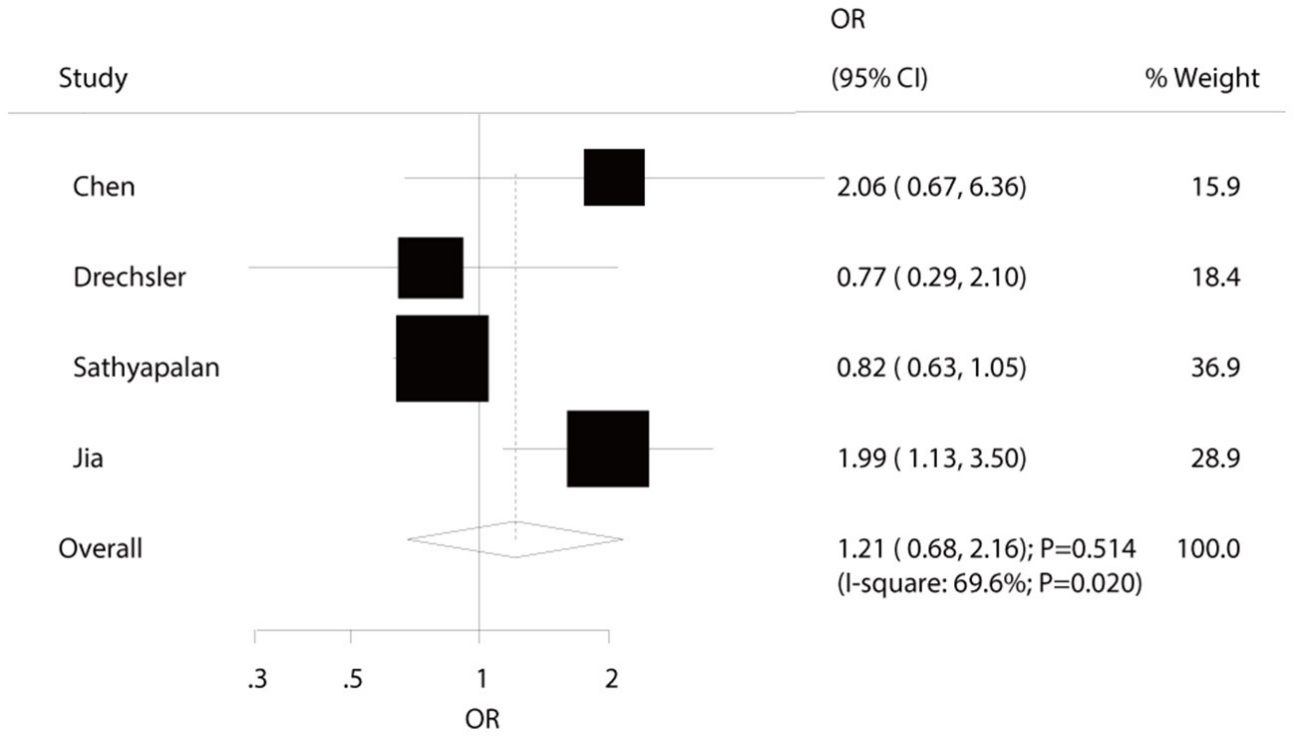
The relation between hypothyroidism and the risk of MACEs in diabetic patients.

Four studies reported an association between hypothyroidism and MACEs risk in diabetic patients. The summary ORs indicated no significant association of hypothyroidism with the risk of MACEs (OR: 1.21; 95%CI: 0.68–2.16; *P* = 0.514; Figure 2) and showed significant heterogeneity among the included studies. The sensitivity analyses indicated that the pooled conclusion showed no changes by the sequential exclusion of each study (Supplemental 1). Subgroup analyses indicated that hypothyroidism was associated with increased risk of MACEs when the study design was cross-sectional (OR: 2.01; 95%CI: 1.21–3.32; *P* = 0.007), the study was conducted in Eastern countries (OR: OR: 2.01; 95%CI: 1.21–3.32; *P* = 0.007), and the percentage of male sex was <50.0% (OR: 1.99; 95%CI: 1.14–3.50; *P* = 0.016) (Table 2). No significant publication bias for MACEs was observed (*P*-value for Egger: 0.400; *P*-value for Begg: 0.734; Supplemental 2).

**Table 2:** Subgroup analyses for major cardiovascular events and all-cause mortality.

**Outcomes**	**Factors**	**Group**	**OR and 95% CI**	***P*-value**	**I^**2**^ (%)**	***P*-value for heterogeneity**	***P*-value between subgroups**
Major cardiovascular events	Study design	Cross-sectional	2.01 (1.21–3.32)	0.007	0.0	0.959	0.007
		Prospective	0.77 (0.29–2.07)	0.605	–	–	
		Retrospective	0.82 (0.64–1.06)	0.128	–	–	
	Country	Western	0.82 (0.64–1.05)	0.109	0.0	0.904	0.002
		Eastern	2.01 (1.21–3.32)	0.007	0.0	0.959	
	Percentage male (%)	≥50.0	0.90 (0.61–1.34)	0.610	19.7	0.288	0.007
		<50.0	1.99 (1.14–3.50)	0.016	–	–	
	DM type	Type 2	1.21 (0.68–2.16)	0.514	69.6	0.020	–
		NS	–	–	–	–	
	Study quality	High	1.21 (0.68–2.16)	0.514	69.6	0.020	–
		Low	–	–	–	–	
All-cause mortality	Study design	Cross-sectional	2.56 (0.66–9.92)	0.174	–	–	0.149
		Prospective	1.56 (1.10–2.21)	0.012	0.0	0.973	
		Retrospective	0.79 (0.29–2.14)	0.646	73.5	0.052	
	Country	Western	1.17 (0.63–2.20)	0.620	64.5	0.060	0.409
		Eastern	1.28 (0.79–2.08)	0.322	21.2	0.260	
	Percentage male (%)	≥50.0	1.15 (0.44–3.03)	0.774	66.0	0.053	0.911
		<50.0	1.26 (0.98–1.63)	0.070	40.5	0.195	
	DM type	Type 2	1.15 (0.44–3.03)	0.774	66.0	0.053	0.911
		NS	1.26 (0.98–1.63)	0.070	40.5	0.195	
	Study quality	High	1.15 (0.44–3.03)	0.774	66.0	0.053	0.911
		Low	1.26 (0.98–1.63)	0.070	40.5	0.195	

### All-Cause Mortality

**Figure 3:**
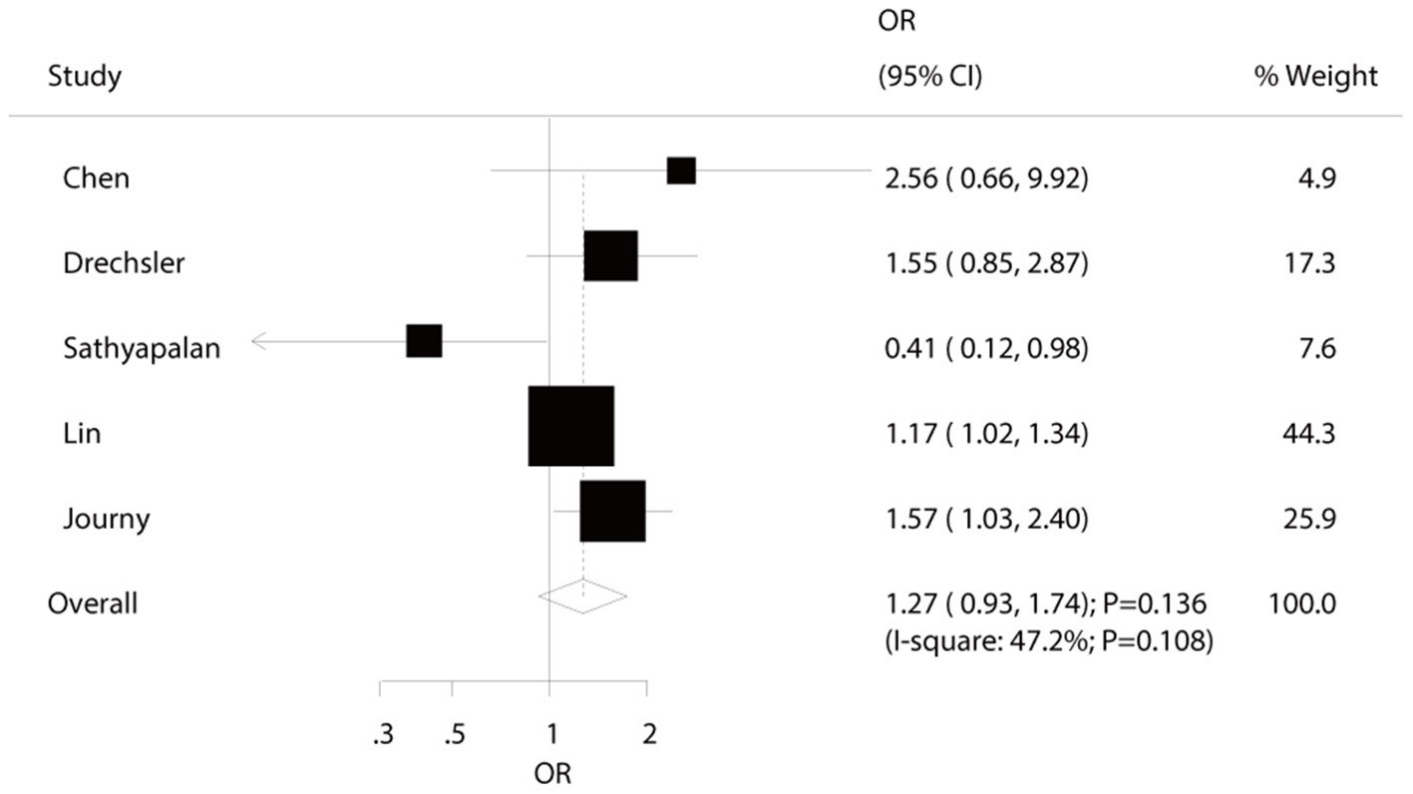
The relation between hypothyroidism and the risk of all-cause mortality in diabetic patients.

Five studies reported an association between hypothyroidism and all-cause mortality risk in diabetic patients. Pooled analysis results indicated no association between hypothyroidism and all-cause mortality (OR: 1.27; 95%CI: 0.93–1.74; *P* = 0.136; Figure 3), and no significant heterogeneity was observed. According to the sensitivity analysis, we excluded the study by Sathyapalan et al. (21), which specifically reported a higher death rate due to other reasons, including 79 respiratory, 18 neoplasm, and six other deaths. After this exclusion, we concluded that hypothyroidism was associated with an increased risk of all-cause mortality (OR: 1.28; 95%CI: 1.06–1.54; *P* < 0.05; Supplemental 1). Moreover, the subgroup analyses indicated a significant association between hypothyroidism and all-cause mortality in pooled prospective cohort studies (OR: 1.56; 95%CI: 1.10–2.21; *P* = 0.012; Table 2). No other significant associations between hypothyroidism and all-cause mortality were observed. No significant publication bias for all-cause mortality was detected (*P*-value for Egger: 0.792; *P*-value for Begg: 1.000; Supplemental 2).

### Other Adverse Events

**Figure 4:**
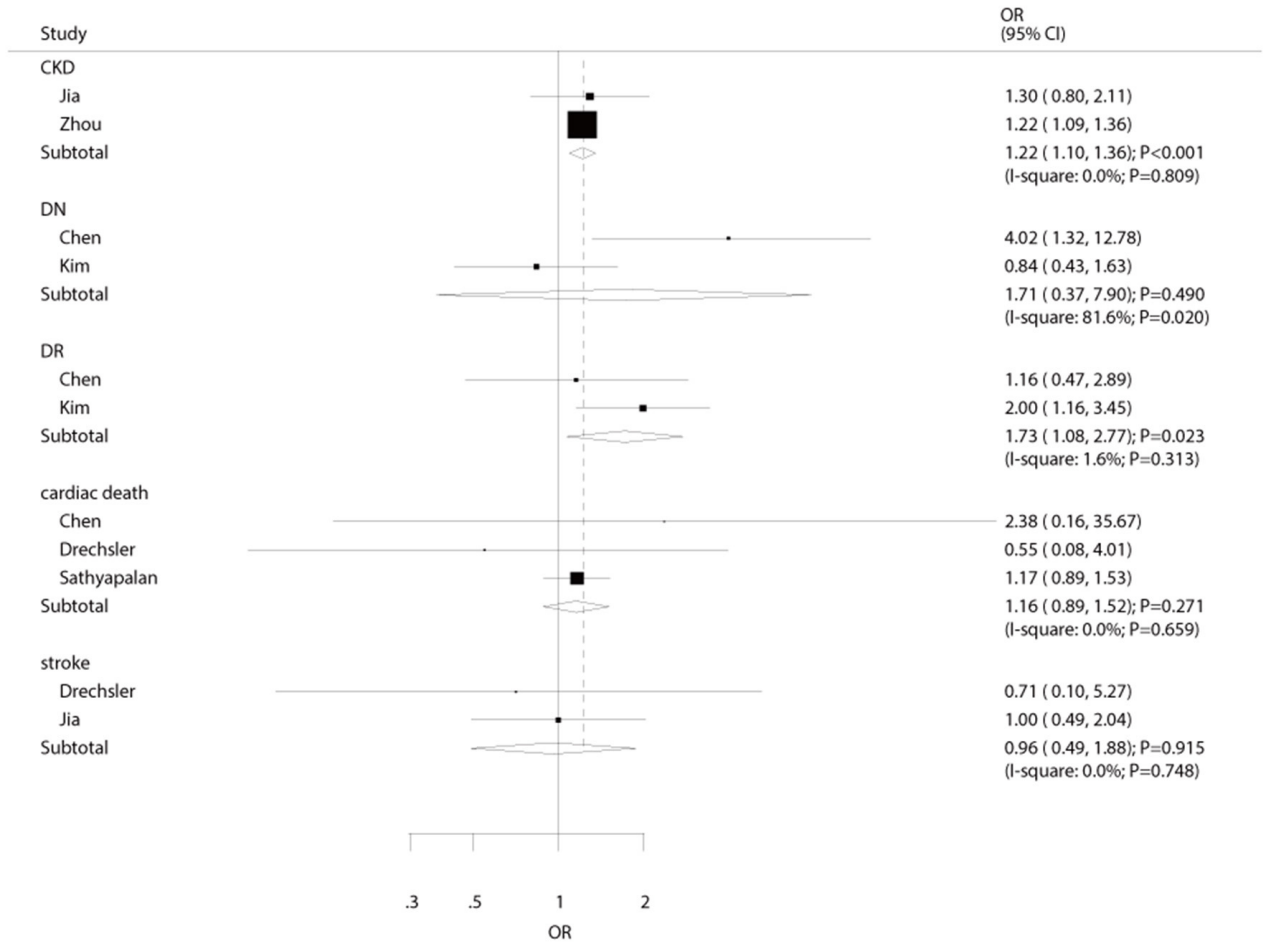
The summary results for the association of hypothyroidism with the risk of cardiac death, stroke, DN, DR, and CKD.

The number of studies available for each outcome was three, two, two, two, and two studies for cardiac death, stroke, DN, DR, and CKD, respectively (Figure 4). We noted that hypothyroidism was not associated with the risk of cardiac death (OR: 1.16; 95%CI: 0.89–1.52; *P* = 0.271), stroke (OR: 0.96; 95%CI: 0.49–1.88; *P* = 0.915), and DN (OR: 1.71; 95%CI: 0.37–7.90; *P* = 0.490). On the other hand, the risk of DR (OR: 1.73; 95%CI: 1.08–2.77; *P* = 0.023), and CKD (OR: 1.22; 95%CI: 1.10–1.36; *P* < 0.001) were significantly increased in diabetic patients with hypothyroidism. Sensitivity, subgroup, and publication biases were not calculated for these outcomes due to the small number of included studies.

## Discussion

The present meta-analysis was conducted based on eight studies that included a wide range of characteristics in terms of study design and patients in order to evaluate the prognostic role of hypothyroidism in diabetic patients. The results of this study indicated that hypothyroidism was associated with greater risks of DR and CKD, whereas no significant association of hypothyroidism was found with the risks of MACEs, all-cause mortality, cardiac death, stroke, and DN. The results of the sensitivity analyses indicated that hypothyroidism might be associated with an increased risk of all-cause mortality when sequentially excluding each study. Subgroup analyses indicated that the relationship between hypothyroidism and MACEs is different based on study design, country, and the percentage of male sex.

Numerous systematic reviews and meta-analyses have already been conducted to explore the potential impact of hypothyroidism on subsequent adverse clinical endpoints. Baumgartner et al. indicated that higher circulating free T4 levels were associated with an increased risk of atrial fibrillation in euthyroid individuals (28). Åsvold et al. found that the differences in thyroid function within the reference range were not associated with the risk of coronary heart disease incidence and mortality (29). Rodondi et al. found that hypothyroidism was associated with an increased risk of coronary events and cardiac death, especially for individuals with TSH levels ≥10 mIU/L (30). A meta-analysis conducted by Han et al. found that type 2 DM is associated with an excess risk of hypothyroidism, and hypothyroidism in diabetic patients results in excess risk of DN, DR, peripheral arterial disease, and diabetic peripheral neuropathy (31). Wu et al. found that hypothyroidism is associated with an increased risk of DR (32). Several other important results (MACEs, all-cause mortality, cardiac death, and CKD) and the results of the stratified analyses for MACEs and all-cause mortality were not conducted. Therefore, the present study was conducted to verify the association of hypothyroidism with adverse clinical endpoints in diabetic patients.

This study indicated no significant association between hypothyroidism and MACEs in diabetic patients, and most included studies (3/4) reported similar results, but the study conducted by Jia et al. indicated that hypothyroidism in patients with type 2 DM was associated with an increased risk of coronary heart disease, while it was not associated with the risk of ischemic stroke (24). They also pointed out that overt thyroid hormone deficiency was associated with higher levels of total cholesterol and LDL-cholesterol. Moreover, it could lead to impaired vascular function, especially in diabetic patients (33–35). Furthermore, the patients with severe hypothyroidism in this study had high total cholesterol and LDL-cholesterol levels, and the mean values exceeded the upper limit of the reference range (24). The subgroup analyses indicated that hypothyroidism was correlated with a higher risk of MACEs if the study had a cross-sectional design, the study was conducted in Eastern countries, and the percentage of male sex was <50.0%. A cross-sectional study cannot provide any evidence of causal relationships between hypothyroidism and MACEs, which might, in turn, overestimate the true relation and biases with uncontrolled factors. Moreover, hypothyroidism in diabetic patients from Eastern countries was diagnosed later, and the upper limit of TSH was higher than in diabetic patients in Western countries. Finally, the percentage of male sex might affect the relationship between hypothyroidism and MACE risk due to differences in lifestyle factors between men and women.

We noted no significant association between hypothyroidism and all-cause mortality in diabetic patients, whereas the results of sensitivity analyses indicated that this pooled conclusion might alter when excluding the study conducted by Sathyapalan et al. (21). The reason for this could be that the study conducted by Sathyapalan et al. reported a high incidence of non-cardiac deaths, and the included patients were older than in other studies (21). Lin et al. found that the risk of all-cause mortality was significantly increased in diabetic patients with hypothyroidism (27), and pointed out that the all-cause mortality might be significantly increased due to the cardiovascular risk, which is considered as the leading cause of death in hemodialysis patients (36). Moreover, Journy et al. reported similar results and indicated a significant positive relationship between hypothyroidism and all-cause mortality (25). The results of the subgroup analyses were consistent with the overall pooled conclusion in all subsets, which might be due to the intrinsic relation between hypothyroidism and all-cause mortality being affected by additional confounders. Although the results of the sensitivity analysis indicated significantly increased risk of all-cause mortality in diabetic patients with lower thyroid function, this significant increase might be overestimated due to two studies that included patients diagnosed with hypothyroidism, but not subclinical hypothyroidism (25, 27).

The summary results showed diabetic patients with hypothyroidism were significantly at higher risk of DR and CKD, while the associations of hypothyroidism with the risks of cardiac death, stroke, and DN showed no significant association. Kim et al. indicated that hypothyroidism was associated with an increased risk of severe DR in patients with type 2 diabetes (22). The study specifically assigned patients who underwent a comprehensive evaluation for DR based on dilated eye examination and fluorescein angiography. Moreover, patients with hypothyroidism had atherogenic disturbances in lipid metabolism, and the relationship between DR and dyslipidemia has already been demonstrated (37, 38). In addition, the study conducted by Zhou et al. pointed out that hypothyroidism in type 2 DM patients was associated with an increased risk of CK (26). The reason for this might be due to the following reasons: (1) thyroid hormone affect kidney growth and function (39, 40); (2) hypothyroidism is associated with low cardiac output, and higher peripheral vascular resistance cause intrarenal vasoconstriction (31); (3) hypothyroidism is correlated with endothelial dysfunction, causing small vessel dysfunction (32); and (4) the altered iodine metabolism decreases peripheral sensitivity to hormone, and autoimmune thyroiditis are significantly associated with chronic inflammation (31). Interestingly, the summary results indicated that hypothyroidism was not associated with the risk of DN in diabetic patients. This result could be due to the study conducted by Kim et al. (22), which reported a lower prevalence of DN owing to patients with renal insufficiency being excluded, and broad 95%CI were obtained, i.e., no statistically significant difference.

There are several limitations that should be acknowledged in this study: (1) several cross-sectional and retrospective studies were included, which might introduce potential uncontrolled biases and cause overestimation of the effect estimates; (2) DM types in two studies were not specified, which might, in turn, affect the prognosis of hypothyroidism; (3) the studies by Lin et al. and Journy et al. did not distinguish between subclinical and clinical hypothyroidism (25, 27); (4) the pooled results for cardiac death, stroke, DN, DR, and CKD were calculated from smaller numbers of included studies, and the pooled results are unreliable, which need further large-scale prospective study verified; (5) the analysis of this study was based on the pooled data and individual data were not available, restricting our study with more detailed analysis; and (6) publication biases might exist as the analysis was based on published articles.

In conclusion, the results of this study indicated that hypothyroidism was associated with excessive risks of DR and CKD in diabetic patients. Moreover, hypothyroidism might affect the risk of all-cause mortality. On the other hand, no significant associations of hypothyroidism with the risks of MACEs, cardiac death, stroke, and DN in diabetic patients were observed. Further large-scale prospective cohort study should be conducted to verify these findings and calculate the potential dose-response curves.

## Author Contributions

SZ and GF conceived and supervised the study. FK and YG designed experiments. FK, YG, and HT performed experiments. PG and JG analyzed data. SZ, FK, and YG wrote the manuscript. SZ and GF made manuscript revisions. All authors reviewed the results and approved the final version of the manuscript.

### Conflict of Interest

The authors declare that the research was conducted in the absence of any commercial or financial relationships that could be construed as a potential conflict of interest.
