# Supplementary material for: Hypothyroidism and Adverse Endpoints in Diabetic Patients: A Systematic Review and Meta-Analysis
Source: Front Endocrinol (Lausanne). 2020 Jan 10;10:889. doi: 10.3389/fendo.2019.00889 (PMC6965024; doi:10.3389/fendo.2019.00889)
Supplement: Supplementary file 1 [file Data_Sheet_1.docx]

**Supplementary Information**

Supplemental 1: Sensitivity analysis for MACEs and all-cause mortality


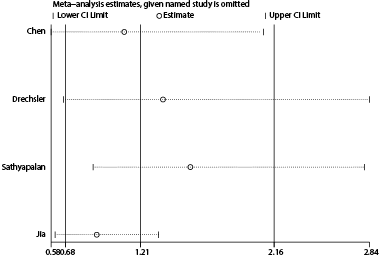


Figure S1. Sensitivity analysis for the relation between SCH and MACEs in diabetic patients


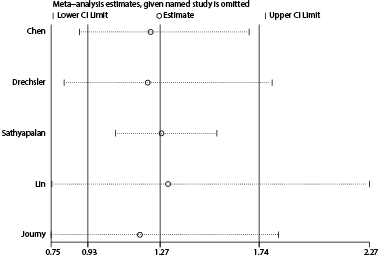


Figure S2. Sensitivity analysis for the relation between SCH and all-cause mortality in diabetic patients

Supplemental 2: Publication biases for MACEs and all-cause mortality


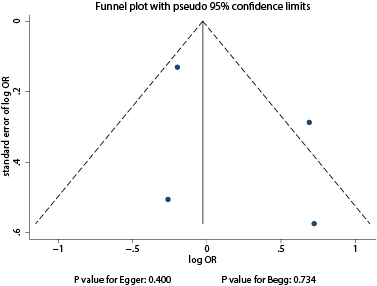


Figure S1. Publication bias for the relation between SCH and MACEs in diabetic patients


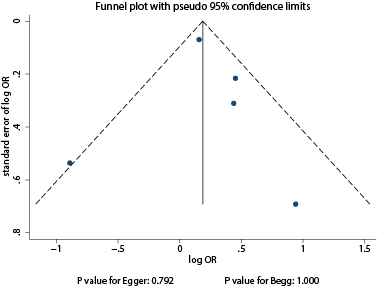


Figure S2. Publication bias for the relation between SCH and all-cause mortality in diabetic patients
